# Slowness of the Pulse without Any Well-Marked Disease to Account for It

**Published:** 1862-01

**Authors:** 


### SLOWNESS OF THE PULSE WITHOUT ANY WELL-MARKED DISEASE TO ACCOUNT FOR IT.

Extreme depression, apoplexy, some organic affections of the heart, and the exhaustion preceding final dissolution, are known to produce slowness of the pulse. By the term *slowness* is meant the diminution of the heart's beat to 40 per minute, or even much lower, say to 30 or 25 beats per minute. Ordinarily the cause of the slow pulse may be made out. Thus, Dr. Watson states in his "Lectures on the Practice of Physic," vol. i. "The slowest pulse I ever felt was that of a man of sixty-eight years old, who was for some time a patient of mine, with diseased heart and dropsy. His pulse was often no more than 25 in the minute. He died suddenly in his chair, and I was very desirous of examining his body, but his widow would not allow it." The same writer refers to a case in the 17th volume of "Duncan's Medical Commentaries," in which the pulse was as slow as 9 beats in a minute.

We have recently watched a case of some interest, in St. Bartholomew's Hospital, under Dr. Farre's care, in which the chief peculiarity was slowness of the pulse. The patient, Wm. C——, a painter by trade, is fifty-eight years of age, and was admitted on the 2d May. Nearly six weeks before admission he was seized with giddiness, accompanied by pain and a sinking sensation in the stomach; he had shortness of breath, and could not walk a distance without stopping. For these he consulted the parochial surgeon, who told him of his slow pulse. Shortly before his admission he had a fainting fit. Whilst in the hospital the pulse was as low as 26 per minute when sitting or lying down, but increased to 30 or 34 when standing. On the 2d June the pulse was 22 per minute; his general health was otherwise good. He remained in the hospital altogether about six weeks, and complained almost solely of debility at the commencement, which gradually disappeared under the influence of a regulated diet and mild tonic remedies. His pulse, though slow, was always regular; there was nothing abnormal about the heart in any way, and his general intelligence was good, although the mind did not appear to be active.

There is then no very decided cause for the slow pulse, unless the symptoms complained of some weeks before admission be interpreted as pointing to some insidious disease of the brain. Although a painter by trade, he has not suffered from the poisoning influences of his trade.—*British American Journal.*

